# Unmasking the appeal – tobacco and vaping companies have recruited a new generation of nicotine dependents

**DOI:** 10.5588/pha.26.0034

**Published:** 2026-05-18

**Authors:** L. Hagen, G.N. Kazi

**Affiliations:** 1 Action on Smoking & Health (ASH Canada), Edmonton, AB, Canada;; 2 Dopasi Foundation, Islamabad, Pakistan.

**Keywords:** tobacco control, tobacco industry, Generation Z, electronic cigarettes, Juul

## Abstract

Tobacco consumption has long been implicated in several lethal diseases. However, the tobacco industry has continued to develop new products and marketing schemes to attract new generations of customers and sustain its market share and sales. This year’s World No-Tobacco Day theme zeroes in on the driving force behind the global tobacco epidemic – the tobacco industry and its relentless pursuit of new victims.

Tobacco consumption in any form has long been implicated in several lethal diseases.^1–3^ While the WHO report on the global tobacco epidemic holds tobacco use responsible for over 7 million deaths annually, as well as disability and long-term suffering,^4^ for decades, the tobacco industry has continued to develop new products and marketing schemes that appeal to youth to attract new generations of customers and sustain its market share and sales. This year’s World No-Tobacco Day theme zeroes in on the driving force behind the global tobacco epidemic – the tobacco industry and its relentless pursuit of new victims. The major target of the tobacco and nicotine industries is Generation Z, those born between 1997 and 2012, and the first generation to grow up in the era of omnipresent online social media.^5^ This was also the first generation in a hundred years to be confronted with a global pandemic that drove youth to online platforms to socialise, conduct their studies, and seek entertainment during COVID-19 lockdowns. The trend towards increased online and digital engagement by youth has not been lost on the tobacco and nicotine industries.

Advertising and media exposure can shape youth perceptions and intentions regarding vaping. Environmental factors are strongly associated with youth vaping intentions, including online engagement through various digital platforms. As Hopkinson et al. point out, youth in the UK who spend 1–3 h per day on social media are over 90% more likely to vape.^6^ The tobacco and nicotine industries have deployed various strategies and campaigns to target youth through popular online platforms, including Instagram, TikTok, and YouTube, and hired media influencers to promote vaping products. The depiction of vaping and vaping products in online entertainment, digital streaming productions, and video games has become commonplace, including promoting their evolving products on online platforms. These industries primarily position nicotine products as safe ‘harm reduction’ alternatives to cigarettes, concealing the fact that nicotine is addictive and harmful, particularly to children and youth.^7^ Indeed, youth who use nicotine vaping products are at least 2–3 times more likely to become smokers, according to three systematic reviews.^8–10^ Adolescents also risk dual use of nicotine and tobacco when they jointly use tobacco and vape to satisfy their growing cravings. Although marketed as non-combustion nicotine delivery devices, the internal heating elements often cause the thermal decomposition of vaping liquids, which include nicotine, glycerine, propylene glycol, flavourings, and other additives.^11^ The emissions produced by this ‘cooking’ process include known human carcinogens such as formaldehyde and benzene, several harmful volatile organic compounds, and PM2.5 particles, which are a standard indicator of poor air quality. Not surprisingly, several review articles have revealed that nicotine vaping among nonsmokers is a significant risk factor for respiratory illness, cardiovascular disease, and cancer, albeit lower than that from tobacco combustion.^12–14^ Nevertheless, extreme caution is warranted to protect youth from ‘trendy’ products crafted for Gen Z, including heated nicotine and tobacco products that provide the full nicotine ‘buzz’, imply less harm, and thereby promote youth experimentation.

The most notorious of these new products is ‘Juul’, which delivers a strong nicotine ‘hit’ in a well-disguised container that could be easily mistaken for a USB memory stick by unwitting parents and teachers. With vaping products continuing to become more youth-friendly, enticing digital displays, music, animations, cartoon characters, flavours, smells, and sounds, nicotine volumes have also increased, with some disposable products delivering up to 100,000 hits/puffs. The enormous commercial success of Juul contributed to the youth vaping epidemic, and Gen Z was a primary target. So profound was the influence of Juul on the youth vaping epidemic that dozens of states in the USA have successfully sued the company for its contributing role.^17^ Juul’s influence on youth vaping has been profiled extensively in the news media, including a 2019 cover story in Time Magazine.^15^ The tobacco and nicotine industries’ relentless assault on Generation Z is a colossal social failure that continues unabated, even a decade after the game-changing launch of Juul. While governments and international agencies like the WHO have taken action to protect youth, many of these measures have fallen short of substantially solving the problem. Policymakers need to confront these industries and implement strong measures that align with successful tobacco control laws to the greatest possible extent. It is also imperative for future health care and global health professionals to have a thorough understanding of the risks associated with all forms of smoking, including e-cigarettes, to advocate for tobacco control along realistic lines.^16^ The foregoing also validates a position statement of the Union from 2015, unequivocally stating that ‘The safety of electronic cigarettes or electronic nicotine delivery systems has not been scientifically demonstrated’.^17^ It also reinforces the Union’s stance almost a decade ago to adopt a holistic approach that effectively addresses the issue from all angles and looks beyond the direct health implications of e-cigarette use to explore the social, economic, political, and environmental aspects of this debate, putting ‘harm reduction’ in context.^18^

The tobacco industry’s fallacious ‘harm reduction’ narrative needs to be unmasked to reveal the ruthless transnational corporations that are aggressively targeting teenagers with highly addictive products that pose considerably more harm than good to youth. Countries with strong tobacco control laws and high tobacco taxes have witnessed significant declines in youth tobacco use. The obvious solution to curtail youth vaping is to apply these highly effective tobacco control measures to vaping products and their use. To do anything less will not be doing justice to current and future generations. The time to act is now!
